# Supplementary material for: Prevalence and factors associated with hypertension among adults with and without HIV in Western Kenya
Source: PLoS One. 2022 Jan 10;17(1):e0262400. doi: 10.1371/journal.pone.0262400 (PMC8746744; doi:10.1371/journal.pone.0262400)
Supplement: Attachment — Submitted filename: PLOS ONE-REVIEWERS COMMENT.docx [file pone.0262400.s001.docx]

**PLOS ONE**

**TITLE: Hypertension prevalence and correlates among adults with and without HIV in Western Kenya**

**Authors:** Mogaka et al,

**General comment:** Mogaka et al investigated prevalence of hypertension and its correlates among adults with and without HIV in Western Kenya. This study is of importance as high blood pressure is on the increase in Africa. The impact of this on individuals living with HIV will be huge hence authors’ effort is highly commendable and appreciated. However, I have the following observations:

1. From the introduction, it was clear that the prevalence of hypertension in Kenya has been well researched and published as shown by references 4,9,10. One therefore wondered on what the essence of this present study aim at achieving is.
2. There is a need for the authors to consider re-writing the introduction as the current one is not catching or appealing enough.
3. Methods: Did authors actually use data from 300 HIV-negative adults (Line 114)? See line 62 of abstract.
4. Line 135: was the blood sample for CD4 T lymphocyte and viral load\ not preserved using anticoagulant? Please throw more light on this.
5. Line 160: How did the authors arrive at the cut off value of >130mg/L for LDL-C (As against the well-known cut off of >100mg/dl)?
6. Statistics: Line 168. How did the authors test for normality of the continuous variables?
7. I suggest that authors should stick to one p value (e.g p <0.05 or p<0.001) for the purpose of discussion of the results.
8. Authors should consider presenting some of the results in figures.
9. Critically looking at the first two lines of discussion (lines 241-242), the message is the same with the first line in 2^nd^ paragraph. (Line 249). This should reviewed.
10. Line 282-290 discussed what was never presented as part of the results. There was nowhere in the results that the blood pressure values of the participants were documented. How do I therefore believe the discussion?
11. The references: The references in this section did not follow any style. I suggest that authors should follow the recommended style by the journal.
12. Finally authors have done a great work and I suggest that they consider my observation as this will improve the quality of this manuscript.
