# Supplementary material for: Prevalence and factors associated with hypertension among adults with and without HIV in Western Kenya
Source: PLoS One. 2022 Jan 10;17(1):e0262400. doi: 10.1371/journal.pone.0262400 (PMC8746744; doi:10.1371/journal.pone.0262400)
Supplement: Attachment — Submitted filename: PLOS ONE REVIEWERS COMMENT-2.docx [file pone.0262400.s003.docx]

**TITLE: Factors associated with hypertension prevalence and correlates among adults with and without HIV in Western Kenya**

**Authors:** Mogaka et al,

**General comment:** Mogaka et al investigated Factors associated with hypertension prevalence and correlates among adults with and without HIV in Western Kenya. This study is of importance as high blood pressure is on the increase in Africa. The impact of this on individuals living with HIV will be huge hence authors’ effort is highly commendable and appreciated.

I have read the revised version of the manuscript. Authors have addressed majority of issues raised. However I am still of opinion that authors should present some of the results in figure. Also all the tables should be properly presented by having only 3 horizontal parallel lines. The current table format does not fit into scientific table presentation.

In the overall, authors have improved the quality of the manuscript.
