# Supplementary material for: Prevalence and factors associated with hypertension among adults with and without HIV in Western Kenya
Source: PLoS One. 2022 Jan 10;17(1):e0262400. doi: 10.1371/journal.pone.0262400 (PMC8746744; doi:10.1371/journal.pone.0262400)
Supplement: Attachment — Submitted filename: Response to Reviewers comments_1st Nov21.docx [file pone.0262400.s004.docx]

Ref. Number:	PONE-D-21-10329

Dear PLOS ONE Editors and Reviewers,

Thank you very much for the opportunity to revise and resubmit our manuscript, entitled ***“Prevalence and factors associated with hypertension among adults with and without HIV in Western Kenya.****”* We have revised our manuscript in response to the comments made by reviewers. In this cover letter, we provide a point-by-point response to the reviewers’ comments. We are returning two copies of the revision, one in which the changes are tracked (“tracked”) and the other in which all changes have been accepted (“clean”).

We greatly appreciate your consideration and look forward to hearing from you.

Sincerely,

Jerusha Mogaka (corresponding author)

**TITLE: Prevalence and factors associated with hypertension among adults with and without HIV in Western Kenya**

**Journal Requirements:**

- Please review your reference list to ensure that it is complete and correct. If you have cited papers that have been retracted, please include the rationale for doing so in the manuscript text, or remove these references and replace them with relevant current references. Any changes to the reference list should be mentioned in the rebuttal letter that accompanies your revised manuscript. If you need to cite a retracted article, indicate the article’s retracted status in the References list and also include a citation and full reference for the retraction notice.

***Response: Thank you for the suggestion. We have reviewed the reference list and found none of the references has been retracted. However, we have replaced line 437 (reference 27) with the current WHO guidelines on physical activity.***

***“****Bull FC, Al-Ansari SS, Biddle S, Borodulin K, Buman MP et al. World Health Organization 2020 guidelines on physical activity and sedentary behaviour. British Journal of Sports Medicine 2020;54:1451-1462. doi.org/10.1136/bjsports-2020-102955”*

**Editorial Comments**

**Reviewer #2**

- I have read the revised version of the manuscript. Authors have addressed majority of issues raised. However I am still of opinion that authors should present some of the results in figure. Also all the tables should be properly presented by having only 3 horizontal parallel lines. The current table format does not fit into scientific table presentation.

***Response: As per your recommendations, we have presented some of our results as a figure (line 228). We have also formatted all tables to reflect a scientific presentation table***

- **Title Re-wording**

After consultation with the authors, we have re-worded the title to remove repetition as *“factors associated”* and *“correlates” are similar terms.*

The title of our manuscript is ***“Prevalence and factors associated with hypertension among adults with and without HIV in Western Kenya.”***
